# Methods of Protecting Buildings against HPM Radiation—A Review of Materials Absorbing the Energy of Electromagnetic Waves

**DOI:** 10.3390/ma13235509

**Published:** 2020-12-03

**Authors:** Krzysztof Majcher, Michał Musiał, Wojciech Pakos, Adrian Różański, Maciej Sobótka, Tomasz Trapko

**Affiliations:** Faculty of Civil Engineering, Wroclaw University of Science and Technology, pl. Grunwaldzki 11, 50-370 Wroclaw, Poland; krzysztof.majcher@pwr.edu.pl (K.M.); wojciech.pakos@pwr.edu.pl (W.P.); adrian.rozanski@pwr.edu.pl (A.R.); maciej.sobotka@pwr.edu.pl (M.S.); tomasz.trapko@pwr.edu.pl (T.T.)

**Keywords:** building, materials, protection, HPM, review

## Abstract

The pulsed high power microwave (HPM) technology has been developed worldwide for over 20 years. The sources of HPM pulses are a weapon of mass destruction. They pose danger especially to computer and telecommunications equipment and systems, both the military and civilian ones. This paper presents a survey of literature on electromagnetic wave radiation absorbing and shielding materials to be used in construction. Relevant protective measures should include the shielding of buildings or their parts and the absorption of radiation by building envelopes and their elements. The main focus is on the possibilities of improving the shielding and absorptive properties of common construction materials, such as concrete, mortars and synthetic resins. The survey covers the following groups of materials: carbon-based admixtures, nickel powder, iron powders, ferrites, magnetite and polymers. The final part of the survey is devoted to hybrid foam microwave absorbers in which the shape of the material’s inner structure and that of its surface play a special role.

## 1. Introduction

This paper presents a survey of knowledge on electromagnetic wave radiation absorbing and shielding materials to be used in building materials providing protection against high power microwave (HPM) radiation. The pulsed HPM technology has been developed worldwide for over 20 years. This applies to both HPM pulses and ways of protecting against them [1,2]. The pulses induce very high currents in electronic circuits, causing their damage or destruction. They pose danger especially to computer and telecommunications devices and systems, both the military and civil ones. The electronics subjected to the action of these pulses become useless as they are damaged or their software freezes [3,4]. The effect of HPM pulses and their possible use are the basis of modern cyber warfare. HPM radiation sources are a weapon of mass destruction, but a weapon that basically does not cause directly human casualties. Nevertheless, one can imagine cases of HPM pulse attacks as a result of which people die, e.g., attacks on civil or military aircrafts. Such an attack can be directed at a selected area, but also at selected targets, in the latter case it is the so-called point attack [1,2,5,6]. As a kind of modern weapon, HPM pulses are characterized by the following [3,4,7]:

- A frequency in the range of approximately 1–100 GHz,
- A high power of the emitted pulses (peak output powers on the order of 1 GW),
- A very short duration of the pulses (10′s–100′s ns).

When designing ways of protecting buildings against attacks with the use of this weapon one should take into account that HPM pulse energy can reach devices and systems through interfaces, loose building envelopes, utility inlets, window and door openings and also through space dividers. An attack on a building can lead to the paralysis of its subsystems. This interference into the building’s control and management systems can result in (besides the measurable losses to the infrastructure) loss of the health and even life of the people evacuated in panic from the endangered building devoid of power supply, ventilation, air-conditioning, etc.

Protection against directed HPM pulse energy needs to be comprehensive, covering both the design and construction of a building. As regards construction, the protective measures should include the shielding of rooms, space enclosures and openings and the absorption of radiation by space enclosures and their components [8,9]. Importantly, the effectiveness of a protection system is determined by its weakest element. Thus, apart from walls, ceilings or a roof, the protection of ventilation openings, doors and windows need to be solved. These problems are discussed, e.g., in [10,11] in the context of windows and in [12] where apertures are considered.

This survey is aimed mainly at improving the shielding and absorptive properties of construction materials, such as concrete, mortars and synthetic resins. Absorptive properties can be usually improved by dispersing suitably designed particles, having high electric conductivity or magnetic properties, in the base material (e.g., concrete). An additional improvement in the absorptive properties is achieved by suitably shaping the material’s surface or its inner structure. The aim of such modifications is to make the material subjected to the HPM pulse attenuate it as much as possible by dissipating its energy converting it into a heat. In general, the attenuation depends on the electrical permittivity (dielectric constant) and magnetic permeability of the material used but also on the frequency of microwaves and on the thickness of the absorbing layer. For a given frequency, the effectiveness of a specific absorber (with a fixed thickness, filler type, its content, form and granulation) is most often determined in the literature by providing a value of either shielding effectiveness or reflection loss. In view of the applications considered in this review, a high level of microwaves attenuation over a wide range of frequency is desirable for the absorbers. Taking this into account, the most complete characteristic of the effectiveness of the absorber would be either shielding effectiveness or reflection loss as a function of frequency in the entire microwave range. Such data, however, are not always available in the literature. Therefore, in this review, it was decided to provide the fullest possible information (in terms of quantitative characterization of microwave absorption), which is given in almost all of the analyzed works, i.e., either shielding effectiveness or reflection loss together with the corresponding frequency range.

Most of this survey deals with microwave radiation absorbing admixtures and their effectiveness, as described in the literature on the subject. In particular, the possible uses of carbon-based materials (Section 2) and the proposed uses of nickel powder (Section 3), iron powder (Section 4), ferrites (Section 5) and magnetite (Section 6) are presented. Section 7 presents hybrid solutions using polymers. Section 8 is devoted to foam microwave absorbers in which especially multiple wave reflection, achieved by appropriately shaping the material’s structure and its surface, is exploited to improve the absorptive properties. The paper ends with concluding remarks presented in Section 9.

## 2. Carbon-Based Materials

So far such carbon-based materials as graphite, coke, carbon fibers, carbon nanotubes, graphene, fly ash and carbon black have been used for protection against electromagnetic pulses.

Graphite in the form of a powder is a highly electrically conductive filler. Tests have shown that the use graphite powder (from 17.5% up by volume) combined with high-alumina cement paste enhances the effectiveness of electromagnetic shielding [13]. This effectiveness increases with the graphite powder percentage content. At the graphite powder content of 30% by volume a 3 mm thick sample showed a shielding effectiveness of 10–40 dB in the frequency range of 200–1600 MHz.

Colloidal graphite can be obtained from very fine graphite powder by introducing the latter into water or alcohol together with a small amount of polyvinyl alcohol (PVA). After application the water or the alcohol evaporates from the solution, whereby the graphite particles come into contact with one another. Tests carried out on samples with a nominal thickness of 4.4 mm and a different graphite content showed that the composite obtained in this way was characterized by high shielding effectiveness [14]. In addition, a sample with a surface colloidal graphite coating was tested. The results reported in [14] are presented in Table 1. The authors of the work investigated the shielding effectiveness of cement paste with graphite content using the transmission line method. It was assumed that the measurements of electrical resistivity would be optimal due to the fact that electrical resistivity is the basic quantity describing the electrical conduction behavior. Thus, the shielding effectiveness was defined in [14] as attenuation upon transmission. The measuring stand consisted of a shielding effectiveness tester (SET 19A by Elgal) with its input and an output connected to the HP8510A network analyzer. While the DC resistivity was measured with a Keithley 2001 multimeter.

Graphite can also be used in the form of the so-called elastic graphite. This material is produced in the form of sheets obtained by pressing graphite flakes. The flakes are joined together through interlocking. Owing to exfoliation, elastic graphite has a large specific surface area (in the order of 15 m^2^/g). Moreover, it is characterized by high conductance (especially in the sheet’s plane). Consequently, it is a material particularly effective in shielding against electromagnetic pulses. Its shielding effectiveness was estimated in [15] to be as high as 130 dB at a frequency of 1 GHz. In study [16] flake graphite electrolessly plated with a FeNi alloy was tested. The minimal reflection losses were found to amount to about −45 dB for a frequency of ca. 12 GHz and a coating thickness of 1.2 mm.

Study [17] presents research in which petroleum coke, containing no less than 99.0% carbon, was used as an admixture to cement paste. The tested cement paste made of Portland cement was characterized by a water–cement ratio (*w*/*c*) of 0.35. The results of the tests [17], in which the petroleum coke percentage by volume was varied, are presented in Table 2. The admixture was found to increase shielding effectiveness.

Another material that can be used as a filler are carbon fibers. Their shielding effectiveness is much higher than that of the coke mentioned above. Fibers less than 0.1 µm in diameter are referred to as filaments [13]. Owing to their slenderness they are more advantageous shielding-wise than fibers [18]. In [19] it was found that carbon filaments 0.1 µm in diameter and not shorter than 100 µm, used in the amount of 0.54% by volume in cement paste, resulted in the shielding effectiveness of 26 dB. This effectiveness can be increased to 30 dB through the use of dispersing agents: methylcellulose (0.4% by cement weight) together with silica fume (15% by cement weight) or latex (20% by cement weight). The use of 3 mm long carbon fibers at their content of 0.84% by volume resulted in the shielding effectiveness of about 15 dB [20]. The above test results were obtained for the radiation frequency of 1.5 GHz.

It is worth noting that the use of a carbon fiber admixture as dispersed fiber reinforcement enhances the mechanical qualities of cement composites, which from the point of view of protecting building structures against HPMs is an additional desirable feature of this filler. In study [21] compression and bending tests were carried out on cement mortar samples and axial tension tests were carried out on cement paste samples. Carbon fibers with the following parameters: length—5 mm, diameter—10 µm, density—1.6 g/cm^3^, Young’s modulus—48 GPa and tensile strength—690 MPa were used. The fibers content by volume amounted to respectively: 0.37%, 0.53% and 0.35% for the three different specimen types: compressive (cube shape), tensile (dog-bone-shaped) and flexural (beam shape). Increments in tensile and flexural strength relative to samples without carbon fiber reinforcement were noted. Other advantages of cement composites with reinforcement of this type include increased resistance to shrinkage and beyond-the-elastic-range behavior, which are not observed in elements without this reinforcement.

In the literature one can also find hybrid solutions based on carbon compounds. An example here is a colloidal graphite coating with carbon filaments [22]. In the case of water-based colloidal graphite the shielding effectiveness amounted to 11.2 dB. After carbon filaments (with a diameter of 0.1 µm and a minimum length of 100 µm) amounting to 35% by volume were added the shielding effectiveness increased to 24.2 dB. The tests were carried out for the frequency of 1 GHz. The advantages of the use of carbon fibers in a composite with a carbon and polymer matrix are described in [23].

Considering their large specific surface area and slenderness, carbon nanotubes seem to be a particularly promising material providing protection against electromagnetic pulses. They are characterized by relatively high strength and elasticity and low density. These properties make nanotubes a very good admixture to other materials [24]. It has been shown that adding even a small quantity of carbon nanotubes one can achieve a percolation threshold at which electrical conductivity, and so shielding effectiveness, significantly increases. In [25] it was found that an addition of multilayer wall carbon nanotubes to high-strength mortar affected the latter’s compressive strength (which increased by 25–58%), impact strength (which increased by 1400%) and three-point bending strength (which increased by 2%). Unfortunately, in [25] the error bars were not given what would have been especially important in the case of so small growth of the three-point bending strength. The above values are for the nanotubes content of 0.1% relative to the cement mass.

Many research on the protection of built structures against microwave radiation, e.g., [25], are devoted to the use of graphene, mainly in the form of cladding, for this purpose. In particular, graphene is used as:

- An ultrathin composite in which graphene is combined with epoxy resins, polystyrene, polyethylene, polyurethane, etc.;
- Three-dimensional porous foam, e.g., formed through the hydrothermal polymerization of a carbon source (a mixture of phenol and formaldehyde) and graphene oxide (GO), chemically activated with potassium hydroxide (to achieve the required conductance and specific surface area) [26];
- Three-dimensional graphene sponge (GS) [27].

Additionally, research on hybrid solutions, e.g., combining graphene and carbon nanotubes, is conducted [28]. Moreover, the effectiveness of Fe nanoparticles encapsulated in carbon nanotubes was tested in [29]. The minimal reflection losses were found to amount to −40 dB for approximately 17 GHz at a layer thickness of 1.5 mm. The absorbing power of an ordered mesoporous C-SiO_2_-Fe nanocomposite dispersed in an epoxy resin matrix was investigated in [30]. Two reflection loss minima, which at the layer thickness of 2 mm were equal to about −35 dB for 13 GHz and −30 dB for 16 GHz, were determined. In the frequency range of 12–16 GHz reflection loss RL <−10 dB. In [31] an innovative method was used to synthesize Ni/carbon foam characterized by low density, high surface area and porosity, moderate conductance and significant magnetism. The foam exhibited high microwave absorbability. The minimal reflection losses amounted to about −45 dB for 13.3 GHz at the layer thickness of 2 mm. Possibilities of the absorption of microwaves (in the range of 2–18 GHz) by composites incorporating activated hollow carbon fibers (ACHFs), carbon nanotubes (CNTs) and Ni nanoparticles were examined in [32]. The results of measurements showed that the CNTs–Ni–ACHFs absorber was more effective than the samples of Ni–ACHFs and ACHFs. The minimum reflection loss for the CNTs–Ni–ACHFs composite amounted to −43.457 dB at 13.10 GHz and the thickness of 2.0 mm.

Fly ash—a byproduct of coal combustion [33]—was examined with regard to protection against electromagnetic interference in paper [34]. Fly ash is commonly used in construction as a component of cements and an admixture to cement composites. It modifies the properties of a concrete mixture (improves its workability) and the concrete (enhances its resistance to an aggressive sulphate environment, slows down concrete hardening, increases heat resistance, reduces shrinkage and can influence water tightness, water absorption, compressive strength and Young’s modulus). Tests carried out on cement paste [34] showed that when the cement was (fully) replaced with fly ash, the shielding effectiveness increased by as much as 100%.

The authors of [35] showed that the absorptive properties of slab absorbers could be improved by randomly distributing carbon black (CB) inclusions in them. Experimental results confirmed the significant improvement of the absorptive properties of such slabs (in a wide range of frequencies) in comparison with conventional absorbing slabs (not containing CB inclusions). In the case of a 30% weight fraction of CB particles, the highest absorption effectiveness was registered in the 9 GHz band.

Table 3 shows the shielding effectiveness or reflection losses of the selected carbon-based materials reported in this chapter. For each material, the frequency range and the corresponding level of shielding effectiveness or reflection losses expressed in dB are given.

It is also worth mentioning the papers [36,37]. The authors considered the problem of the shielding effectiveness with the use of the reverberation chamber. In the tests [36] the shielding effectiveness of carbon nanotube reinforced concrete composite was studied. The weight percentages of carbon nanotubes were 0, 1 and 3 wt % and the frequency band was 0.8–8 GHz. The authors proved that 30 mm thick reinforced concrete composite with 3 wt % of the nanotubes provides a shielding effectiveness of more than 15 dB for frequency about 2 GHz and up to 30 dB for 8 GHz. In the paper [37] the electromagnetic absorbing cross section (ACS) of carbon foams in the mm-wave frequency range 50–70 GHz was tested. As the authors mentioned, the reverberation chamber method allows one to study the electromagnetic interaction with materials by taking into account not a single wave propagation mode but rather a homogeneous and statistically random electromagnetic field propagation.

## 3. Solutions Based on Nickel Powder

The research works cited in this section indicate high potential for the use of nickel in creating novel composite materials in the form of various powders, films and electrolytic coatings covering base materials. Materials in which nickel performs the role of an addition (e.g., a coating) show an increase in electromagnetic wave absorption in the range of microwave frequencies in comparison with the base material without nickel. Composite materials in which nickel powder is a primary ingredient deposited in the matrix (e.g., a polymeric matrix) are good absorbers of microwaves owing to the high magnetic permeability of the nickel, whereby such materials absorb more electromagnetic pulses than most metals.

**Figure 1:**
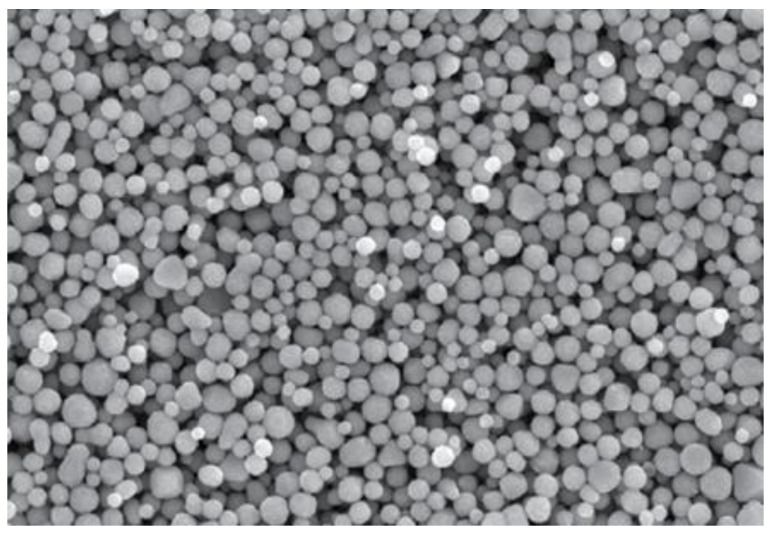
Ultrafine nickel powder with a grain diameter of 0.2 µm [38].

The properties of nickel powder produced in the carbonyl process to a large extent depend on the conditions in which the nickel carbonate decomposes and on the further processing. With regard to grain size, the nickel powders currently produced for the broadly understood industry are classified as follows:

- Ultrafine—with a grain diameter of 0.1–0.4 μm (Figure 1),
- Light—with a grain diameter of 2–4 μm,
- Heavy—with a grain diameter of 4–10 μm,
- Nickel pellets—with a grain diameter of 5–10 mm.

According to Zhonghao Lu et al. [39], ultrafine nickel powder is a promising material for electromagnetic waves absorption owing to its very high magnetic transformation temperature. As a result, its waves absorption capacity remains stable in a wide range of temperatures (practically it cannot deteriorate). Shielding and absorptive properties to a large extent depend on the distribution of powder particles in the base material. According to the authors of [40], due to nickel’s electrical properties combined with its oxidation resistance nickel powder could be used to shield electronic equipment against electromagnetic interference (EMI). Even though nickel is not as good a conductor as other metals (e.g., copper and silver), owing to its high magnetic permeability it absorbs more EMI than most metals. The electromagnetic absorption properties of a composite consisting of an ideally reflecting metal surface coated with a nickel-zinc ferrite (Ni_0.65_Zn_0.35_Fe_2_O_4_) film were examined in [41]. It was demonstrated that in the case of a large coating thickness such a composite was a relatively good electromagnetic absorber in the frequency range of 1 MHz–1.5 GHz and was characterized by a medium reflected energy value (below 20%). In [42] the microwave absorptivity of hybrid nickel powders with Ti_3_SiC_2_ was tested in the range from 8.2 to 12.4 GHz. It was shown that nickel powder enrichment with Ti_3_SiC_2_ significantly contributed to an increase in the dielectric loss of such powders, whereas their magnetic loss decreased in comparison with pure nickel powder. According to the authors of [42], advantageous microwave absorption properties can be obtained by varying the Ti_3_SiC_2_ fraction content. A composite with an epoxy resin matrix, containing 30% Ni and 30% Ti_3_SiC_2_, was characterized by optimal microwave absorbance. In the case of the 2.2 mm thick composite, a reflection loss (RL) below 10 dB within the frequency range of 8.2–12 GHz was obtained.

Highly promising results of research into microwave absorption by nickel powders differing in their particle size are reported in [43]. Monodisperse nickel micro- and nanopowders were produced using the chemical reduction of the aqueous solution of NiSO_4_, NaOH and NaH_2_PO_2_. The influence of pH and the initial NiSO_4_ concentration on the grain size, structure, morphology and microwave absorption properties of several powders was examined. The powders differed in their grain size determined by X-ray diffraction (XRD). The microwave absorbance of the composite materials based on the nickel powders was determined using a network analyzer. The nickel powders were shown to absorb microwaves in the wide frequency range of 0.5–18 GHz. In [44] the authors using electroless deposition coated SiO_2_ particles with a Ni-Co-P layer. The composite powder obtained in this way was subjected to an analysis covering its morphology (electron microscope scanning), crystal structure (XRD), surface structure (X-ray photoelectron spectroscopy) and magnetic and microwave absorbance (using a vibrating sample magnetometer and a network analyzer). Ultrafine Ni-Co-P-SiO_2_ composite powder was found to exhibit very high microwave absorbance. The maximum microwave loss of the 3.10 mm thick protective coating amounted to 48.6 dB at the frequency of 4.2 GHz.

In study [45], where nickel nanostructures were successfully deposited on graphene nanolayers through direct electrochemical plating, it was shown that such a combination exhibited good electromagnetic wave damping effectiveness. The nickel nanostructures content and morphology and the magnetic properties of the graphene alone and those of the graphene coated with nickel were analyzed by means of, i.a., a vibrating sample magnetometer. The relative magnetic permeability and permittivity of the graphene and the material with nickel-coated graphene were measured. Reflection loss values were calculated for the range of 2–18 GHz according to electromagnetic wave propagation theory. In comparison with the graphene, the material with graphene coated with nickel exhibited significantly better electromagnetic wave absorption properties [36]. For example, when the absorbing layer was 1 mm thick, the maximum absorbance of the graphene amounted to about −6.5 dB at the frequency of about 7 GHz. In the material with nickel-coated graphene at the adsorbing layer thickness of 1.5 mm, absorbance exceeded –10 dB within the 5 GHz band while the maximum absorbance amounted to −16.0 dB at 9.15 GHz. A very promising electromagnetic wave absorbing material, i.e., a reduced graphene oxide/NiO composite (RGO/NiO), was proposed in [46]. A 3.5 mm thick coating layer exhibited a reflection loss not less than −55.5 dB at 10.6 GHz. Furthermore, within the bandwidth from 10.2 to 16.9 GHz, the absorption effectiveness amounted to 90% (RL = −10 dB).

**Figure 2:**
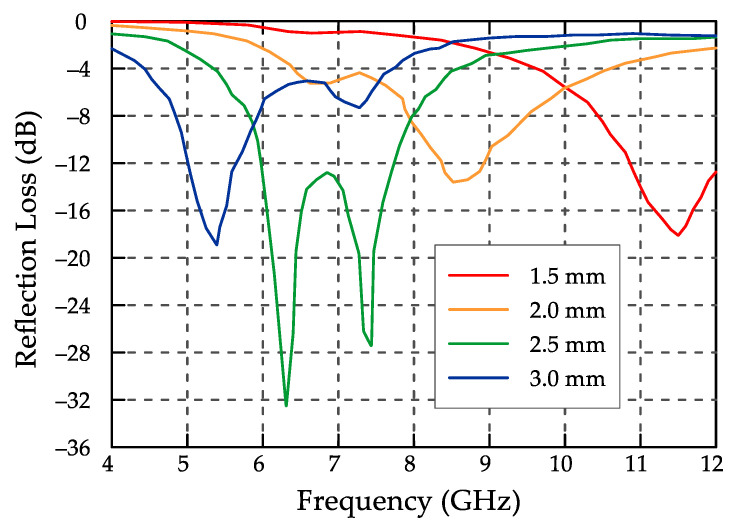
Reflection loss versus frequency for SiC powders coated with Ni-Co-P at different sample thicknesses (based on the data presented in [47]).

The authors of study [47] focused on silicon carbide coated with Ni-Co-P. Silicon carbide particles coated with Ni-Co-P were produced by two-step (step 1—pre-treatment and step 2—coating) electroless deposition and were to be used as a light microwave absorber. The properties of the composite were verified using the same methods as in [44,45]. The test results showed that owing to the conductive and ferromagnetic behavior of the thin nickel-cobalt (Ni-Co) layers both a high dielectric constant and magnetic loss can be obtained in the range of microwave frequencies. The measured maximum microwave loss of the composite powder amounted to about 32 dB at the frequency of 6.30 GHz and the thickness of 2.5 mm, while the initial atomic ratio of Ni to Co in the electrolytic bath amounted to 1.5. The graph of the reflection loss as a function of the frequency (limited to the spectrum range between 4 and 12 GHz) is presented in Figure 2 at various sample thicknesses.

Table 4 shows the reflection losses of the nickel powder-based materials reported in this chapter. For each material, the frequency range and the corresponding level of reflection losses expressed in dB or % are given.

## 4. Iron Powders

Shield magnetic fillers, such as iron powders (and also ferrites, described further on), mainly generate magnetic losses and so are responsible for the absorption of the magnetic component. The usefulness of iron powders in absorptive composites was indicated in [48], where the influence of the size of iron powder particles on the microwave absorption properties of composites was studied. The optimal (absorption-wise) size of Fe powder particles was determined using analytical methods and through experimentation. When the particle size was increased relative to the optimal size, this would result in a sharp decrease in the absorbing capacity of the investigated composites.

Carbonyl iron (CI) powder and carbon black were used to produce layered composites in [49]. The two-layered materials were developed in order to increase the electromagnetic wave absorbing capacity of slabs and cladding. The structure of CI and CB was analyzed using a scanning electron microscope and a scanning transmission electron microscope. Additionally, the dielectric properties and the absorption mechanism were analyzed. The results obtained within the frequency range of 2–18 GHz show that the double-layer absorber is characterized by two absorption peaks and the location of the peaks changes depending on the particular component content. If, e.g., the CI fraction by weight amounts to about 50% and the total absorber thickness is equal to 4 mm, the effective absorption (below −8 dB) band reaches 5.5, 5.8 and 6.5 GHz. When the CB fraction by weight amounts to 50% or 60% and the CI fraction by weight amounts to 70%, the effective absorption band does not exceed 10 GHz (below −4 dB). In study [50] it was confirmed that the effectiveness of absorbers can be increased through the use of isolated CB particles dispersed in a water-based varnish matrix.

A material in which carbonyl iron particles were deposited on graphite using the chemical vapor decomposition (CVD) method was investigated in [51]. In the frequency range of 2–18 GHz the minimum reflection loss was found to amount to −25.14 dB and −26.52 dB for the layer thickness of 6 and 8 mm, respectively.

Microwave radiation absorbing particles having a carbon fiber (CF) core enveloped with a carbonyl iron (CI) shell are described in [52]. In comparison with composites made of solely carbon fibers, this solution results in a significant improvement in microwave radiation absorbance. The reflection loss for a sample with the CF/CI ratio equal to 1:8.8 exceeded −10 dB. The tests were carried out in the frequency range of 2–18 GHz for the absorber thickness of 0.9–3.9 mm. The minimum reflection loss amounted to −21.5 dB and corresponded to the frequency of 6.6 GHz and the thickness of 2.0 mm.

Table 5 shows the reflection losses of the iron powder-based materials reported in this chapter. For each material, the frequency range and the corresponding level of reflection losses expressed in dB are given.

## 5. Ferrites

As mentioned in the previous section, ferrites are used to protect against radiation mainly due to the fact that they cause magnetic losses. Owing to this they can be used as magnetic fillers in shields. Generally, ferrites are ceramic materials exhibiting ferromagnetic properties and in regards to their electrical properties, they are semiconductors. Ferrites are usually produced by sintering metal oxides (Fe_2_O_3_, MnO, NiO, MgO, CuO, ZnO, SrO and BaO) in suitable proportions. Ferrite particles are used as ingredients in composite panels, cladding and shielding providing protection against electromagnetic interference [2,6,53]. Mainly spinel ferrites or hexaferrites are used as magnetic fillers in such composites [54]. As shown in [55,56], in comparison with spinel ferrites, hexaferrites with planar magnetic anisotropy seem to be an interesting solution in terms of electromagnetic energy dissipation.

Due to their relatively low cost, low density, high electrical resistance and high microwave magnetic loss, barium ferrite powders are quite commonly used. For example, in study [57] barium ferrites were used to create single- and double-layered absorbers and their absorption capacity was determined. The double-layered absorber was found to have a much wider absorption band than the single-layer absorber. The thickness of the two absorbers was almost equal, amounting to 3.6 mm and 3.7 mm, respectively.

The good absorptive properties of layered structures were also indicated in [58]. It was shown that the absorptive properties of ferrites could be improved by forming the latter as layered ferrite–ferrite or ferrite–dielectric systems. By combining ferrites differing in their composition and thickness one can significantly improve their absorptive properties.

The very good absorptive properties of barium ferrites are also mentioned in [59]. Additionally, the effect of chromium (Cr) and aluminum (Al) admixtures on the absorptive properties of ferrites was examined there. When Fe^3+^ was partially replaced with Al^3+^ and Cr^3+^, the absorbance of the barium ferrites significantly improved. It was found that their ferromagnetic resonant frequency changed. It was shown that the multimodal character of the ferromagnetic resonance increased the microwave absorption capacity of the barium ferrites.

The authors of [60] devised a way of producing barium-based ferrite powders. The powder was mixed with epoxy resin to produce a microwave absorbing material. Similarly as in [57], the absorptive properties of single- and double-layered absorbers differing in their layer thickness were compared. The usefulness of such absorbers for military purposes was indicated. Studies [57,61] clearly demonstrate the very good absorptive properties of barium ferrites.

The microwave absorbance of a double-layered composite with a cement matrix and Mn-Zn and silica fume inclusions was analyzed in [62]. It was unambiguously concluded that such composites could be used for the production of building materials.

**Figure 3:**
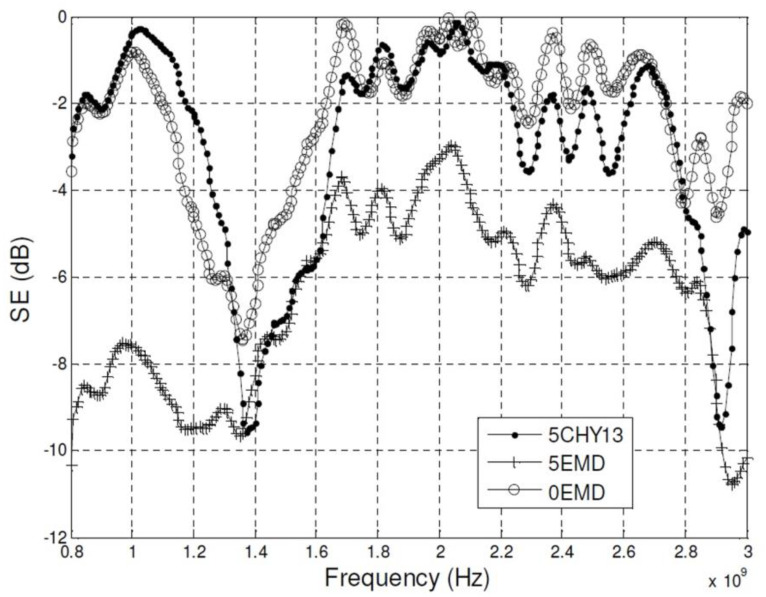
Shielding effectiveness versus frequency for cement plaster containing only sand and for plasters containing manganese-zinc ferrite powders (sample 5CHY13) and manganese dioxide powders (sample 5EMD) [63].

The absorptive properties of cement plasters containing manganese-zinc ferrite powders (sample 5CHY13) or manganese dioxide powders (sample 5EMD) are compared in [63]. The composite’s cement/sand/ferrite or manganese dioxide weight ratio was 1/1/5. The reference sample contained, besides cement, only sand at the weight ratio of 1/6 (sample 0EMD). The authors showed that the introduction of both ferrite and manganese dioxide improved the absorptive properties of the plasters in the frequency range of 0.8–2.8 GHz, but the plaster containing manganese dioxide exhibited better shielding effectiveness (Figure 3).

## 6. Magnetite

Magnetite (Fe_3_O_4_) is a material with a high content of inverse spinel structures in which oxygen atoms are arranged in a closed regular structure with iron atoms in the tetra- and octahedral positions. This structure endows magnetite with ferromagnetic and semiconductor properties. The main advantage of magnetite is that it naturally occurs in the Earth’s crust, whereby its price is relatively low and so as a filler it can be used to industrially produce relatively cheap composite absorbers of electromagnetic waves.

**Figure 4:**
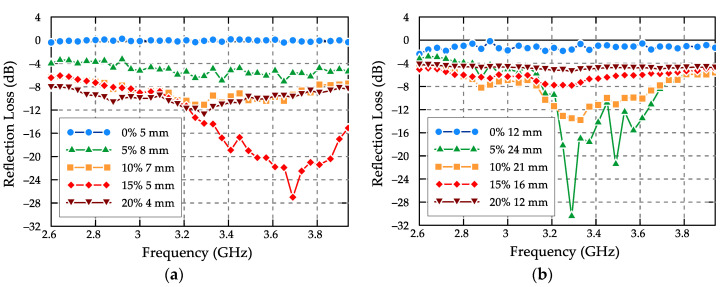
Reflection loss versus frequency at different weight contents of magnetite in cement-based composite (the data extracted from the plots presented in [64]): (**a**) at thin matching thickness and (**b**) at thick matching thickness.

The production and absorptive properties of Portland cement-based composites with a filler in the form of natural magnetite at its weight content ranging from 5% to 20%) were described in [64]. Investigations of the electromagnetic properties of the produced systems in the frequency range of 2.6–3.95 GHz showed that due to the use of magnetite the dielectric and magnetic losses were increased, whereby the absorptive properties of the materials improved. The lowest reflection loss minimum, i.e., −28 dB at 3.7 GHz, characterized the composite with a 15 wt % magnetite content at a thickness of 5 mm. When the magnetite content was increased to 20% by weight, the absorptive properties deteriorated—probably due to the worse impedance fit. The plots of the reflection loss as a function of the frequency at different weight contents of magnetite are presented in Figure 4.

In order to limit the oxidation of Fe_3_O_4_ (characterized by low oxidation resistance), methods of coating magnetite particles with protective layers of nonmagnetic substances have been developed. Most often silica SiO_2_ and less often TiO_2_ and Al_2_O_3_ are used for this purpose [65]. By coating a magnetic particle with SiO_2_ the original properties and morphology of its inner layer (core) are preserved while the properties of its surface change (providing protection against oxidation, etc.) [66]. Moreover, the silica addition increases durability [67], improves strength [68] and accelerates hydration reactions [69]. In study [70] it was shown that due to the SiO_2_ shell a BiOBr (bismuth bromide oxide) particles addition was stably and uniformly dispersed in the cement matrix.

A method of producing core–shell magnetite particles with an outer siliceous (Fe_3_O_4_@SiO_2_) layer is described in [71]. Due to the use of Fe_3_O_4_@SiO_2_, cement particles are better dispersed in the cement matrix and the water absorption rate was lower by 45% in comparison with cement without magnetite particles. The samples were tested in the microwave radiation frequency ranges of 6–18 GHz and 25–42. The content of Fe_3_O_4_ particles with and without the silica (Fe_3_O_4_@SiO_2_) coating in the cement amounted to 3.5% by weight. The minimum reflection loss for the cement with Fe_3_O_4_ amounted to −16 dB at 15.1 GHz and to −22.6 dB at 14.0 GHz.

Magnetite can also be used for filling polymers. In study [72] a polypropylene-natural rubber matrix composite filled with synthetic magnetite was shown to have good absorptive properties. Liquid rubber obtained by photodegrading natural rubber was used as the compatibilizer.

Table 6 shows the reflection losses of the magnetite-based materials reported in this chapter. For each material, the frequency range and the corresponding level of reflection losses expressed in dB are given.

## 7. The Use of Polymers

The possibility of using polymers, especially polyaniline (PANI), for the production of microwave absorbers is mentioned in numerous research. A graphen@Fe_3_O_4_@PANI@TiO_2_ sandwich structure was proposed in [73]. The minimum reflection loss of −41.8 dB for about 14 GHz at the layer thickness of 1.6 mm was obtained. In [74] quaternary composites consisting of reduced graphene oxide (rGO), polyaniline (PANI) and FeNi_3_@SiO_2_ (FeNi_3_ nanocrystals encapsulated in SiO_2_) were found to be excellent electromagnetic wave absorbers. The research showed that the FeNi_3_@SiO_2_@rGO–PANI composites exhibited a wide absorption bandwidth and enhanced the electromagnetic wave absorption properties. The maximum reflection loss reached −40.18 dB for 14.0 GHz at the thickness of 2.4 mm. In [75] polymer nanocomposites prepared by the in situ polymerization of aniline with graphite oxide (GO), γ-Fe_2_O_3_ and BaTiO_3_ were indicated as promising shielding materials. The nanocomposites exhibited a significantly improved electromagnetic shielding efficiency and better thermal properties. The PANI/GO/γ-Fe_2_O_3_/BaTiO_3_ nanocomposite exhibited a shielding efficiency >32.5 dB at the frequency of 1–3 GHz. In [76] the microwave absorption properties of reduced graphene oxide-magnetic porous nanospheres-polyaniline composites were investigated. The minimum reflection loss of the synthesized rGO/porous nanospheres Fe_3_O_4_/PANI composite was found to amount to about −30 dB for approximately 15 GHz at the layer thickness of 1.0 mm. In [77] supermagnetic Fe_3_O_4_/graphene/PANI nanoparticles were studied. The tested sample suspended in paraffin (50/50% by weight) exhibited the minimum reflection loss of about −45 dB for the frequency of 11 GHz and the layer thickness of 2.5 mm.

Table 7 shows the shielding effectiveness or reflection losses of the polymer-based materials reported in this chapter. For each material, the frequency range and the corresponding level of shielding effectiveness or reflection losses expressed in dB are given.

## 8. Hybrid Structured Composites

The microwave radiation absorbing additions discussed in the previous sections constitute the basis for producing effective absorbers applicable in the construction industry. A separate group of materials protecting against HPM is distinguished in this section. They are mostly finished products (panels) in which suitably dispersed admixtures (most of which have already been discussed in this paper) have been combined with a special shape of the absorber’s outer surface and/or inner structure.

**Figure 5:**
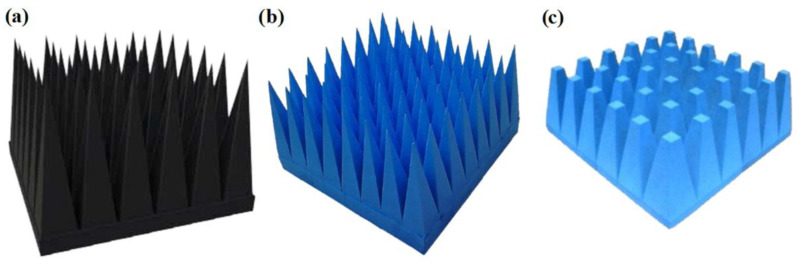
Pyramidal foam absorbers [80]: (**a**) type 5796, (**b**) type 3680 and (**c**) type 3660.

One of the most effective solutions is rubber foams impregnated with carbon and/or iron mixtures [78,79]. They are usually shaped as pyramids arranged close to one another to form a panel [80] (Figure 5). The panel’s height (measured from the pyramid’s base to its apex) is chosen on the basis of the lowest expected frequency at which the assumed amount of energy is absorbed. For the absorption of low-frequency electromagnetic waves this height amounts to about 60 cm while for high-frequency waves it ranges from a few to twenty centimeters.

Radiation absorbent material (RAM) panels are very often used in anechoic chambers [81] in which electromagnetic compatibility (EMC) tests are conducted. The walls of such chambers are covered with panels with their pyramid apexes facing the chamber’s interior. RAM panels damp electromagnetic signals due to two factors: scattering and absorption. The absorber effect occurs both when the reflected waves are in phase and out of phase. The aim of the pyramidal shape is to increase the number of reflected waves since with each reflection the energy of the waves decreases, whereby the signal entering the foam structure is much weaker.

According to [82], in the frequency range of 30–1000 MHz ferrite plates are very good absorbers. They are usually in the form of flat panels made of a ferritic material. However, in comparison with pyramidal panels, they are less effective and so they are usually used when they can be fixed to well-conducting surfaces.

There are also hybrid absorbers [80], combining the advantages (e.g., ease of installation and light weight) of ferritic panels with those (e.g., a wider frequency range of damped signals) of pyramidal panels. Then at a small pyramid height (less than 10 cm) one gets an optimal frequency range in which the signal is effectively absorbed.

Foam absorbers usually consist of fire-resisting polyurethane foam doped with conducting carbon (carbonyl iron and/or crystal graphite) particles amounting to 0.05–0.1% of the total material mass. Further improvements are achieved through a layered arrangement of the conducting particles consistently with the gradient of material density. This means that the pyramid’s apex has the lowest percentage of conducting particles while its base contains the largest number of such particles. As a result, a gentle change in impedance, commensurate with the incoming electromagnetic waves, is achieved and the reflection (echo) is reduced as well. Absorption in a foam material takes place as a result of the conversion of electromagnetic signal energy into heat in the conducting particle. This needs to be taken into account in the case of foam absorbers intended for damping high-power signals as the released heat may require an additional heat removal system (radiators, ventilators, etc.). Currently, there are many producers of ultra-wideband panel foam and/or hybrid absorbers, e.g., [80,82,83], which can be used to protect the walls, floors and ceilings of anechoic chambers, and also to protect satellites and military targets.

Study [84] presents the results of research on foamed concrete with an admixture of organic inclusions. This composite was obtained by adding a foam blowing agent together with animal proteins to Portland cement. For the assumed water-cement ratio (*w*/*c* = 0.4) cement paste was prepared to which foam blowing agent SY-F30 was added at the solution ratio of 1:10. A compressed-air foam generator was used for foaming. Controlling the foam content in the concrete mixture, foamed concrete samples varying in their pore content were produced. It was shown that electromagnetic waves can be absorbed as a result of multiple reflections in the porous medium and that the thickness of the barrier and the material filling ratio have a significant bearing on the wave absorption properties in the frequency range of 2–18 GHz. The widest frequency range with the reflection loss coefficient below −10 dB was obtained for a 40 mm thick sample containing 52.1% (by volume) of foam blowing agent SY-F30. The concrete sample with the foam blowing agent content of 46.7% was characterized by the lowest reflection loss (−34.9 dB) at the frequency of 10 GHz.

**Figure 6:**
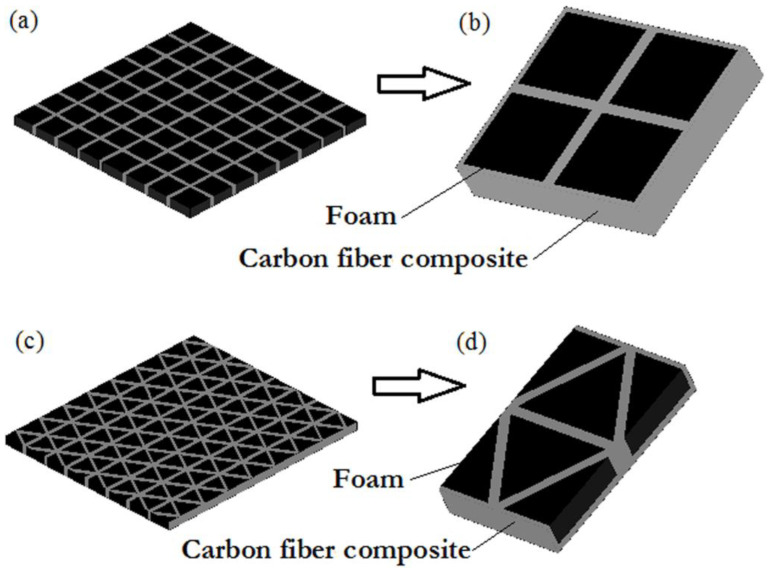
Models of carbon-fiber lattice panels filled with foam absorber: (**a**) rectangular panel, (**b**) rectangular panel model, (**c**) equilateral panel and (**d**) equilateral panel model.

The authors of [85] proposed to fill carbon-fiber lattice structures (currently used in, e.g., aviation) with foam (Figure 6). These are lightweight structures, which when filled with a foam absorber can provide effective protection against microwaves. The study presents numerical analysis results obtained using the spatial network method. The results clearly show that lattice panels filled with a foam absorber while remaining lightweight have the ability to absorb electromagnetic waves at the level of −25 dB in the test frequency range of 8–12 GHz.

Using lyophilization and carbothermal reduction the authors of [86] obtained three-dimensional elastic foams consisting of reduced graphene oxide (rGO) and local SiC nanotube inclusions. The inclusions improved the composite’s electromagnetic wave absorption capacity. It was shown that the novel composite foam absorbers are thermally stable even at the temperature as high as 630 °C. Effective absorbing capacity in the X band (8.2–12.4 GHz) was obtained at a smaller panel thickness (3 mm) than that of ordinary graphene foam.

An interesting concept of a foam microwave absorber, mimicking the natural structure of a honeycomb, was presented by the authors of [87]. Polyurethane foam filled with iron carbonyl or nickel fibers and metal micropowder with magnetic properties was placed in a matrix with a honeycomb-like structure. In this way a composite panel with improved strength (due to the honeycomb structure), at the same constituting a broadband (3–18 GHz) absorber, was obtained. The front side of the panel was additionally coated with titanium nanopowder mixed with rubber composite, which further increases electromagnetic wave attenuation.

Additionally, worthy of noting is study [88] in which the authors present a foam microwave absorber made of graphene foam enriched with polyaniline (PANI) tubes. The reported results show the microwave absorption effectiveness of the GF/PANI composite was much higher than that of the absorber made of undoped graphene. The maximum reflection coefficient amounted to 52.5 dB at the frequency of 13.8 GHz. The composite panel’s thickness ranged from 1.5 to 4 mm.

Table 8 shows the reflection losses of the hybrid structured composites reported in this chapter. For each material, the frequency range and the corresponding level of reflection losses expressed in dB are given.

## 9. Conclusions

It appears from the above survey of knowledge on electromagnetic wave energy absorbing materials with regard to their potential use in construction that protection against high power microwave (HPM) energy can consist of modifying building materials by admixing them with suitably designed particles conducting electricity or having magnetic properties. It is worth noting that the highest protection effectiveness in a wide frequency range can be achieved through the simultaneous use of several admixtures and/or the use of hybrid admixtures, i.e., ones produced from several different materials. A further improvement in the absorptive properties of the finished product is obtained through the suitable shaping of its surface or inner structure.

As regards admixtures in the form of microwave energy absorbing fillers, the possibilities of using materials based on respectively carbon, nickel powder, iron powder, ferrites and magnetite were discussed. Moreover, the potential use of polymers for producing composite admixtures was considered.

Concerning the usefulness of carbon-based materials for protection against electromagnetic pulses the literature indicates the possible use of, i.a., graphite, coke, carbon in the form of fibers and nanotubes, graphene, fly ash and carbon black.

The survey indicates high potential for the use of nickel in creating novel composite materials. Materials modified with a nickel admixture are good absorbers of microwaves owing to the high magnetic permeability of nickel, whereby the latter absorbs more electromagnetic pulses than most metals. Similarly, the use of nickel in the form of a coating covering the base material results in the increased absorption of electromagnetic waves in the range of microwave frequencies in comparison with the uncoated base material.

The use of iron powder as a filler results in merely a moderate microwave absorption level. However, the use of hybrid solutions based on iron powder gives much better results. For example, the hybrid solution in which carbonyl iron particles were deposited on graphite [51] resulted in very good microwave absorption effectiveness. A similar effect was obtained in the case of the composite in the form of a carbon fiber core enveloped with a carbonyl iron shell [52].

Ferrites varying in their composition are very often used to create materials absorbing microwaves. The most commonly used binders are: cement pastes, resins, plastic and rubber [60]. Therefore, ferrites can be used to produce modified structural concrete. Owing to their relatively low cost, low density, high electrical resistance and high microwave magnetic loss, barium ferrite powders [57,58,59,60,61] are most frequently mentioned as highly applicable in practice. In study [57] attention was drawn to the usefulness of such absorbers for military purposes.

The peculiar structure of magnetite endows this material with ferromagnetic and semiconductor properties. Magnetite occurs naturally in the Earth’s crust, whereby it is relatively easily available and inexpensive. Therefore if it is used as a filler, it will be possible to industrially produce relatively inexpensive composite absorbers of electromagnetic waves. Due to the weak oxidation resistance of magnetite, methods of coating its particles with protective layers made of nonmagnetic substances to limit oxidation have been developed. Very good results are obtained when silica SiO_2_ is used for this purpose [65]. Silica also improves the dispersal of magnetite particles in the matrix [71].

Numerous papers also mention the possibility of using polymers, especially polyaniline (PANI), for the production of microwave absorbers. Polyaniline is most often used as a component in hybrid admixtures, usually combined with graphene and/or magnetite [73,74,75,76,77]. The shielding effectiveness of such hybrid material is very high, usually exceeding the reflection loss of −40 dB already at small layer thickness (up to about 2.5 mm).

Materials that besides suitably dispersed admixtures include a specially shaped outer surface or inner structure were discussed as a separate group of materials protecting against HPM radiation. Most often these are finished products manufactured from hybrid panel microwave absorbers. One of the most effective solutions is rubber foams impregnated with mixtures of carbon and/or iron [78,79], usually formed in the shape of pyramids. Study [85] presented a concept of producing an absorber in the form of a panel in which carbon-fiber lattice structures were filled with foam. The absorptive properties of concrete can also be improved by adding foam blowing agents to the concrete mixture. The fact that electromagnetic waves can be absorbed as a result of multiple reflections in a porous medium is exploited in this case. Moreover, the thickness of the barrier and the material filling ratio significantly influence the absorbance of electromagnetic waves.

As regards the modification of construction materials aimed at improving their absorptive properties, besides choosing a filler material, one should take into account the fact that the size of the particles, their amount, their distribution in the matrix and their shape (grains, fibers, flakes, etc.) play a highly significant role. Using smaller filler particles one can more easily (at a lower filler content) achieve a percolation threshold at which conductance, and so shielding effectiveness, significantly increased (e.g., [24,48]). In the case of construction materials, it is also necessary to satisfy the proper requirements concerning stiffness, strength, durability, etc. This means that when creating new construction materials protecting against HPM pulses, one must simultaneously conduct tests to confirm that the technical requirements for the product are met as specified by the proper building codes.

It should be clearly emphasized that the solutions discussed in this survey usually yield a good shielding effectiveness within a relatively narrow frequency range. In order to develop a technology that would comprehensively protect buildings against HPM it is necessary to ensure a proper level of protection in a wide range of electromagnetic wave frequencies. This probably can be achieved only by simultaneously using several partial solutions acting effectively in appropriate frequency ranges. The present authors are currently developing such a system, in the form of a hybrid multilayer wall, and they will soon publish the results of this research.

## 10. Future Development Perspectives

Taking advantage of the present review and the experience gained by the authors from the ongoing works in the project acknowledged in the funding section the following general remarks can be formulated. A methodology of developing construction materials for the purpose of protecting buildings against HPM may consist of the following steps: (1) a testing program in small scale whose results would bring the information on promising absorbers that can be added as fillers to cement based materials; (2) preparation of the composites and tests in the large scale; (3) developing a hybrid solution (e.g., multilayer wall) by combining a few different individual methods of protection; (4) getting approvals, certificates, etc., and (5) implementation of the production in the industrial scale.

The most challenging in steps (1) and (2) is that apart from the microwave shielding performance properties, the materials being developed must meet the requirements for building materials in terms of strength, stiffness, chemical stability, durability, heat conductivity, frost resistance, etc. In addition, the anticipated production costs have to be in mind already at these early stages. However, the costs are not easy to determine because the price of the final product is not only influenced by the unit price of the filler. In addition to the content of the additive, the thickness of the absorbing layer, the possibility of mass production of the additive itself and the necessary technological measures to disperse it in the target material have to be also taken into account.

Furthermore, it needs to be emphasized that the final product or technology must be comprehensive. As mentioned in the Introduction, the weakest element determines the effectiveness of the system. Thus, for instance, a special mortar must be prepared for the bricks or blocks. Moreover, all the elements of a developed technology have to be compatible with each other.

Depending on the type of object protected against HPM, the optimal method of protection needs to be adjusted. For example, for an underground facility, equipped with EM-sensitive electronic devices (e.g., supercomputers, servers, etc.), a method using radiation absorbing materials (RAM) added to structural concrete seems to be sufficient enough providing that installation connections or ventilation are properly protected. For other objects, it may be desirable to hide the function of HPM protection, i.e., to make the building appear like a typical one. Then, a helpful additional element apart from high performance structural elements like walls and ceiling would be the suitably developed windows, doors, gypsum boards, paints, sealings or thermal insulation with an additional function of HPM passive protection.

## Figures and Tables

**Table 1:** Shielding effectiveness of tested samples with colloidal graphite.

Graphite Content by Volume (%)	Layer Thickness (mm)	Shielding Effectiveness (dB)	
		1.0 GHz	1.5 GHz
0	4.36	4.00	2.42
0.46	4.37	10.31	12.34
0.92	4.40	22.31	25.61
coating	0.34	14.26	15.33

**Table 2:** Shielding effectiveness of tested samples containing petroleum coke.

Graphite Content by Volume (%)	Layer Thickness (mm)	Shielding Effectiveness (dB)	
		1.0 GHz	1.5 GHz
0	4.36	4.00	2.42
0.51	4.38	43.5	44.6
1.02	4.41	47.3	49.2
3.06	4.35	48.2	50.2
6.12	4.66	49.3	51.6
9.18	4.77	49.7	51.9

**Table 3:** Shielding effectiveness or reflection losses of carbon-based material.

Material	Shielding Effectiveness (SE)/Reflection Losses (RL)	Frequency/Frequency Range
Graphite powder [13]	SE = 10–40 dB	200–1600 MHz
Elastic graphite (sheet) [15]	SE = 130 dB	1 GHz
Graphite electrolessly plated with FeNi alloy [16]	RL = −45 dB	12 GHz
Carbon fibers [19,20]	SE = 15–30 dB	1.5 GHz
Water-based colloidal graphite [22]	SE = 11.2 dB	1 GHz
Colloidal graphite coating with carbon filaments [22]	SE = 24.2 dB	1 GHz
Fe nanoparticles encapsulated in carbon nanotubes [29]	RL = −40 dB	17 GHz
Mesoporous C-SiO_2_-Fe nanocomposite [30]	RL = −35 dB	13 GHz
	RL = −30 dB	16 GHz
Ni/carbon foam [31]	RL = −45 dB	13.3 GHz
CNTs–Ni–ACHFs composite [32]	RL = −43.5 dB	13.1 GHz

**Table 4:** Reflection losses of nickel powder-based material.

Material	Reflection Losses (RL)	Frequency/Frequency Range
Metal surface coated with a nickel-zinc ferrite film (Ni_0.65_Zn_0.35_Fe_2_O_4_) [41]	RL < 20%	1 MHz–1.5 GHz
Hybrid nickel powders with Ti_3_SiC_2_ [42]	RL < 10 dB	8.2–12.4 GHz
Monodisperse nickel micro- and nanopowders [43]	RL < 12 dB	0.5–18 GHz
Ultrafine Ni-Co-P-SiO_2_ composite powder [44]	RL < 48.6 dB	4.2 GHz
Graphene coated with nickel [45]	RL = −10 dB	5 GHz
	RL = −16 dB	9.15 GHz
Reduced graphene oxide/NiO composite [46]	RL = −10 dB	10.2–16.9 GHz
Silicon carbide coated with Ni-Co-P [47]	RL = −32 dB	6.3 GHz

**Table 5:** Reflection losses of iron powder-based material.

Material	Reflection Losses (RL)	Frequency/Frequency Range
Carbonyl iron powder and carbon black (layered composite) [49]	RL = −8 dB	5.5–6.5 GHz
Carbonyl iron particles deposited on graphite [51]	RL = −26 dB	2–18 GHz
Carbon fiber core enveloped with a carbonyl iron [52]	RL = −21.5 dB	6.6 GHz

**Table 6:** Reflection losses of magnetite-based material.

Material	Reflection Losses (RL)	Frequency
Portland cement-based composite with magnetite [64]	RL = −28 dB	3.7 GHz
Cement with Fe_3_O_4_ [71]	RL = −16 dB	15.1 GHz
	RL = −22.6 dB	14 GHz

**Table 7:** Shielding effectiveness or reflection losses of polymer-based material.

Material	Shielding Effectiveness (SE)/Reflection Losses (RL)	Frequency/Frequency Range
Graphene@Fe_3_O_4_@PANI@TiO_2_ (sandwich structure) [73]	RL = −41.8 dB	14 GHz
FeNi_3_@SiO_2_@rGO–PANI composite [74]	RL = −40.18 dB	14 GHz
PANI/GO/γ-Fe_2_O_3_/BaTiO_3_ nanocomposite [75]	SE > 32.5 dB	1–3 GHz
Synthesized rGO/porous nanospheres Fe_3_O_4_/PANI composite [76]	RL = −30 dB	15 GHz
Supermagnetic Fe_3_O_4_/graphene/PANI [77]	RL = −45 dB	11 GHz

**Table 8:** Reflection losses of hybrid structured composites.

Material	Reflection Losses (RL)	Frequency/Frequency Range
Rubber foams impregnated with carbon and/or iron mixtures [78,79]	RL = −10 dB	1–2.5 GHz
Radiation absorbent material panels [81]	RL = −30 dB	4–18 GHz
Ferrite tile absorber [82]	RL < −10 dB	30–1000 MHz
Wide-band hybrid pyramid EM absorbers [80,82,83]	RL < −10 dB	30 MHz–18 GHz
Polyurethane foam based hybrid absorbers [80,82,83]	RL < −10 dB	30 MHz–18 GHz
Foamed concrete with an admixture of organic inclusions [84]	RL = −34.9 dB	10 GHz
Carbon-fiber lattice structures with foam [85]	RL = −25 dB	8–12 GHz
Elastic foams with reduced graphene oxide and local SiC nanotube inclusions [86]	RL < −10 dB	8.2–12.4 GHz
Polyurethane foam filled with iron carbonyl or nickel fibers and metal micropowder with magnetic properties [87]	RL < −10 dB	3–18 GHz
Graphene foam enriched with polyaniline tubes [88]	RL = −52.5 dB	13.8 GHz
